# Experimental Investigation of a Multicylinder Unmodified Diesel Engine Performance, Emission, and Heat Loss Characteristics Using Different Biodiesel Blends: Rollout of B10 in Malaysia

**DOI:** 10.1155/2014/349858

**Published:** 2014-08-04

**Authors:** M. J. Abedin, H. H. Masjuki, M. A. Kalam, M. Varman, M. I. Arbab, I. M. Rizwanul Fattah, B. M. Masum

**Affiliations:** ^1^Centre for Energy Sciences, Faculty of Engineering, University of Malaya, 50603 Kuala Lumpur, Malaysia; ^2^Department of Mechanical Engineering, University of Malaya, 50603 Kuala Lumpur, Malaysia

## Abstract

This paper deals with the performance and emission analysis of a multicylinder diesel engine using biodiesel along with an in-depth analysis of the engine heat losses in different subsystems followed by the energy balance of all the energy flows from the engine. Energy balance analysis allows the designer to appraise the internal energy variations of a thermodynamic system as a function of ‘‘energy flows” across the control volume as work or heat and also the enthalpies associated with the energy flows which are passing through these boundaries. Palm and coconut are the two most potential biodiesel feed stocks in this part of the world. The investigation was conducted in a four-cylinder diesel engine fuelled with 10% and 20% blends of palm and coconut biodiesels and compared with B5 at full load condition and in the speed range of 1000 to 4000 RPM. Among the all tested blends, palm blends seemed more promising in terms of engine performance, emission, and heat losses. The influence of heat losses on engine performance and emission has been discussed thoroughly in this paper.

## 1. Introduction

The rising oil prices and concerns on the depletion of fossil fuel reserves have forced researchers to not only look into engine optimization, but also find alternative resources to tackle the energy crisis. Biodiesel has gained a growing interest as one of the most promising solutions. Its primary advantages are biodegradable, renewable, and carbon neutral and the fact that it does not produce hazardous toxic gases [1–3]. Another major advantage of biodiesels is that they can be used either pure or blended with fossil diesel fuel at any proportions and can be burnt in the existing diesel engines without any further modifications [4].

### 1.1. The Reasons for Choosing Only These Feed Stocks and Blends

The main (about 73%) agricultural product of Malaysia is oilseeds, mostly palm oil. According to United States Department of Agriculture (USDA), Malaysia is the world's second largest producer (32.7%) and exporter (40%) of palm oil behind Indonesia (production-53.3% and export-49.5%) [5]. One of the reasons for choosing palm and coconut oil is that they are abundant in this particular region. For example, rapeseed in European Nations and soybeans in USA are abundant. Countries like Malaysia, Indonesia, and Thailand, and so forth, have vast costal area with surplus palm oil and coconut oil. Besides, in terms of oil productivity these feed stocks are on the top [6].

Palm oil based B5 biodiesel first rolled out in central regions of Malaysia at June 1, 2011, and then nationwide in early 2013. B5 is now available at 247 BHPetrol stations in Kuala Lumpur and consumes 1.03 million litres of palm-oil biodiesel in each month, which saves nearly 12.4 million litres of fossil diesel fuel consumption per year [7]. According to Malaysian Plantation Industries and Commodities Minister, “B10 will be launched nationwide as soon as the new standard is established and will phase out B5.” They are formulating action plans with the help of Malaysian Palm Oil Board, which includes engine performance, emission, thermodynamics, and tribology analysis of B10 or higher blending ratio as required by the Original Equipment Manufacturers, especially on engine warranty [8].

### 1.2. Literature Review

In a 6-cylinder diesel engine [9], at full load and constant (1500 RPM) speed using palm biodiesel, it was reported that the power is decreased by 2.5%, the BTE is decreased by 0.48%, and the BSFC is increased by 7.5%. The article also reported lower CO (86.89), HC (14.29%), and smoke (67.65%) emission and higher NO_*x*_ (22.13%) emission compared to diesel fuel. In a four-cylinder diesel engine [10] at full load and different engine speeds for various palm biodiesel blends, the power is almost the same for 10% biodiesel and decreased with the increase of further blend ratio. They also reported lower BTE and higher (max. 11%) BSFC compared to diesel fuel. That article found lower CO, HC, and smoke emission and higher NO_*x*_ emission for palm blends. Another article [11] reported similar results for coconut biodiesel in a four-cylinder diesel engine under similar engine conditions. However, according to single cylinder diesel engine reports, the results are similar (lower BTE and higher BSFC) for palm [12, 13] and coconut [14, 15] biodiesels though a few exceptions were also found [16, 17]. Similar emission results were reported for single cylinder engines, but few researchers [18–20] reported slightly lower NO_*x*_ emission while operated on palm and coconut biodiesel. Almost all researchers reported that the lower calorific value, higher kinetic viscosity and density (which cause poor fuel spray and atomization) of biodiesels are responsible for lower brake power and higher BSFC.

Energy balancing was reported for biodiesel [21, 22]. But those are not comprehensive and mostly focused on other topics. They reported that all the heat losses except exhaust heat loss were higher while using biodiesels compared to diesel fuel. This was attributed to the promotion of better combustion of biodiesel fuels. The reason for lower exhaust heat loss was attributed to the lower concentration of HC and CO emission in the exhaust gas for biodiesel fuels and it decreases more with the increase of biodiesel percentage in the blends [21].

### 1.3. Objectives

The objectives of our experiment are to analyze the variations in engine performance and emissions as well as in heat loss characteristics when we go beyond 5% biodiesel (B10 and B20) using palm and coconut biodiesel.

## 2. Experimental Setup and Procedure

**Figure 1:**
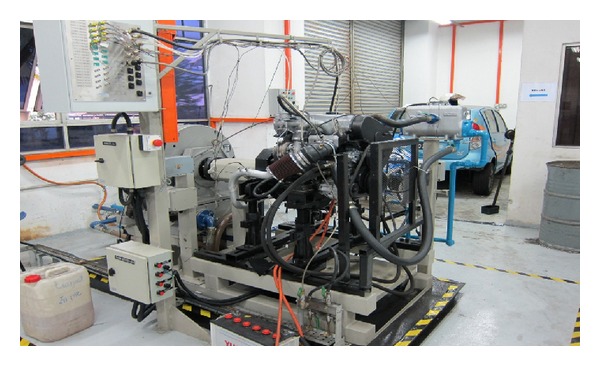
Picture of the engine test bed.

The experiment was performed on a Mitsubishi-4D56 diesel engine. Engine test bed picture is provided in Figure 1 and a detailed engine specification is given in Table 1.

The engine was loaded with a water cooled passive eddy current dynamometer. Therefore the test bed was connected to the data acquisition board, which collects signal, rectify, filter, and convert the signal to the data to be read. The data acquisition board is connected to the laptop, where user can monitor, control, and analyze the data using software through REO-dCA controller. It provides full test sequence control with automatic and manual data logging options. The transducer box provides 16 K/J thermocouple input channels, where we have used a total of 8 k-type thermocouples at 8 different locations to measure cooling water, lubricating oil, and exhaust temperatures. It also provides the facility of fuel flow, oil flow, and air flow measurements by using different pressure transducers. Rotameters have been used to measure engine cooling water flow.

In our experiment, we prepared four different biodiesel blends, that is, 10% and 20% of each biodiesel. The palm blends are presented as P10 and P20 and the coconut blends are presented as C10 and C20, respectively, where the numerical value denotes biodiesel percentage in the blend. The engine was operated at full throttle condition and varying speeds from 1000 to 4000 RPM at an interval of 500 RPM. Crude palm and coconut oil were provided by Forest Research Institute Malaysia (FRIM). B5 biodiesel was brought by the authors. The oils were converted to the biodiesels and properties have been measured in the authors' energy laboratory and engine tribology laboratory-1st floor, Department of mechanical Engineering, UM, 50603 KL, Malaysia. The measured fuel properties of all tested fuel blends are listed in Table 2 along with equipment specifications.

The exhaust emissions of our experiment were measured by using BOSCH (BEA-350) exhaust gas analyser. The specification of the analyser is provided in Table 3.

## 3. Theory of Heat Loss Calculation

**Figure 2:**
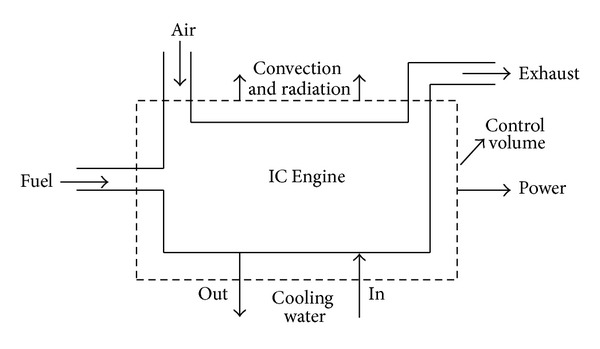
IC engine control volume showing energy flows [2].

From Figure 2, if we consider the IC engine as a control volume (surrounded by control surface), then the energy flows from and to the engine can be expressed by the equations as follows.

The steady flow 1st law of thermodynamics for this control volume will be [23]

(1) $$\begin{array}{c} Q_{s}=P_{b}+Q_{w}+Q_{\text{oil}}+Q_{\text{exh}}+Q_{\text{un}}, \end{array}$$

where *Q*_*s*_ is the supplied fuel energy and is given by

(2) $$\begin{array}{c} Q_{s}={\dot{m}}_{f}\times Q_{\text{LHV}}, \end{array}$$

where ${\dot{m}}_{f}$ and *Q*_LHV_ are the mass flue rate and calorific value of the fuel, respectively.

The engine brake power (*P*_*b*_) can be computed by

(3) $$\begin{array}{c} P_{b}=2\times \pi \times N\left(\text{rev}/\text{s}\right)\times T\left(N\cdot m\right)\times 10^{-3}, \end{array}$$

where *N* and *T* are the engine speed and torque, respectively.

The cooling water heat loss (*Q*_*w*_) can be calculated by using the following equation:

(4) $$\begin{array}{c} Q_{w}={\dot{m}}_{w}\times C_{w}\times \Delta T_{w}, \end{array}$$

where $\dot{m_{w}}$ and *C*_*w*_ are the mass flow rate and specific heat of water, respectively, and Δ*T*_*w*_ is the temperature difference between the cooling water inlet and outlet.

Now, if we can measure the required heat to increase the temperature of the total mass (air + fuel) from the outside air temperature (*T*_*a*_) to the exhaust gas temperature (*T*_*g*_), then we can compute the exhaust heat loss (*Q*_exh_) from the engine. The specific heat of air at mean exhaust gas temperature is assumed as the average specific heat (*C*_*g*_) of the exhaust gases for exhaust heat loss calculation [24]. So, the equation for exhaust heat loss calculation will be

(5) $$\begin{array}{c} Q_{\text{exh}}=\left({\dot{m}}_{f}+{\dot{m}}_{a}\right)\times C_{g}\times \left(T_{g}-T_{a}\right). \end{array}$$

Like cooling water heat loss, the lubricating oil heat loss (*Q*_oil_) can be found by using

(6) $$\begin{array}{c} Q_{\text{oil}}={\dot{m}}_{\text{oil}}\times C_{\text{oil}}\times \Delta T_{\text{oil}}, \end{array}$$

where $\dot{m_{\text{oil}}}$ and *C*_oil_ are mass flow rate and specific heat of lubricating oil, respectively, and Δ*T*_oil_ is the temperature difference between the lubricating oil inlet and outlet. Finally, the unaccounted heat loss (*Q*_un_) can be estimated by applying subtraction rule

(7) $$\begin{array}{c} Q_{\text{un}}=Q_{s}-\left(P_{b}+Q_{w}+Q_{\text{exh}}+Q_{\text{oil}}\right). \end{array}$$

## 4. Results and Discussion

### 4.1. Performance Characteristics

This section will provide an in-depth analysis of the impact of biodiesel blends on engine brake power, fuel consumption, and thermal efficiency, at full load condition and varying engine speeds.

#### 4.1.1. Engine Brake Power (*P*_*b*_)

**Figure 3:**
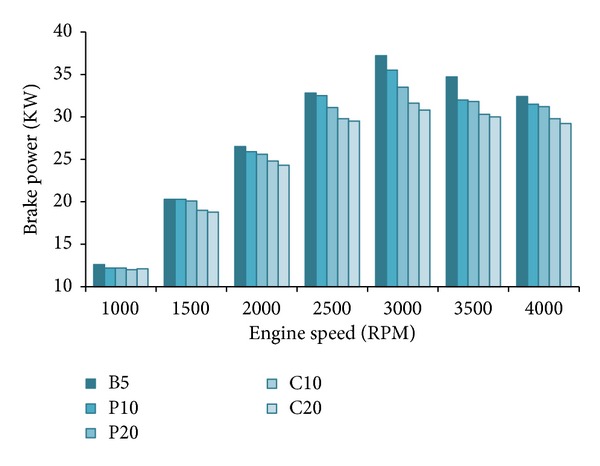
Brake power (*P*_*b*_) at full load.

Figure 3 shows the variation of brake power (*P*_*b*_) of the tested biodiesel blends with varying engine speeds at 100% load. The brake power is increased steadily with increasing speeds to a maximum point for all fuel blends then falls down slightly at the end. B5 showed maximum brake power and palm blends showed close to B5 throughout the speed range. Coconut biodiesel has the lowest calorific value and the highest viscosity among the all blends. As expected, they showed poor performance comparing to others. Researchers [3, 25, 26] attributed the reasons to the lower calorific value and higher viscosity of biodiesel blends. According to them, the excess oxygen content of biodiesels lowers their calorific values. Lower calorific value and higher viscosity cause uneven combustion hence lower brake power [27].

#### 4.1.2. Brake Specific Fuel Consumption (BSFC)

**Figure 4:**
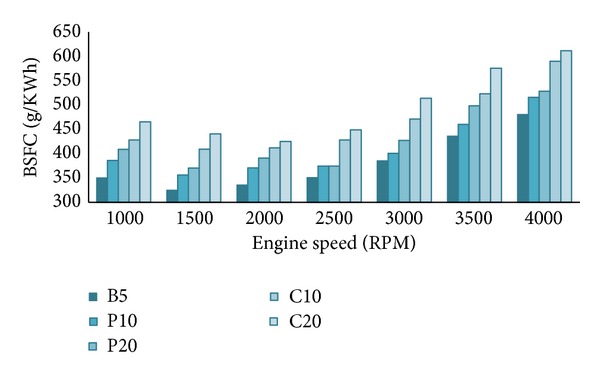
Variation of BSFC for all tested blends at 100% load.

Figure 4 shows the nature of BSFC for all tested fuel blends with varying engine speeds. Fuel properties like density, viscosity, and calorific value clearly influence BSFC [28]. For example, lower calorific value means more fuel needs to be burned in the combustion chamber for the same power output. Again higher kinematic viscosity of biodiesels may cause poor atomization of the fuel, hence poor mixing with air and hence higher BSFC [29]. Clearly, the C10 and C20 blends exhibited highest BSFC throughout the speed range. B5 and palm blends showed lower BSFC compared to others which supports the given explanation. BSFC increased with the increase of biodiesel percentages in the blends. Similar results were found by other researchers [3, 18, 25].

#### 4.1.3. Brake Thermal Efficiency (BTE)

Brake thermal efficiency appraises how efficiently an engine can transform the supplied fuel energy into useful work. Most of the supplied fuel energy will be loss as heat with the engine cooling water, lubricating oil, and exhaust gas [30]. This will be explained in detail in the heat loss section. For instance, we found that biodiesel increment increases engine frictional losses which dissipates through the engine coolant, cylinder walls, and so forth, which means lower BTE for biodiesel increment.

**Figure 5:**
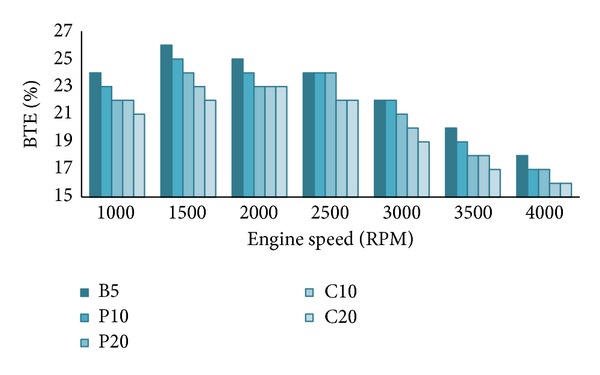
Brake thermal efficiency of all tested fuel blends at full load and various speeds.

As expected BTE decreases with the increase of biodiesel percentage in the blends. BTE is inversely proportional to BSFC and fuel calorific value [4]. We already know that calorific value decreases and BSFC increases with increasing biodiesel percentage. But the BSFC increment is much more dominant here that is why BTE decreases for biodiesel increment despite the lower calorific value. From Figure 5, B5 showed highest BTE among all tested blends and P10 is close to it. At full load condition, fuel consumption increases with the increasing engine speed; hence the BTE decreases [30]. Most of the researchers [4, 10, 12, 14, 29] reported similar results but a few researchers [16, 31] found reverse trend. The reason for higher BTE was attributed to the better combustion of oxygen rich biodiesels [21].

### 4.2. Emission Characteristics

At present, the governments are much more concerned about environmental pollution. Besides, they have imposed strict emission regulations for automobiles. So, the emission analysis of biodiesels is very important before their practical implementation. In this section, the influence of biodiesel blend ratios and engine speeds on gaseous emissions like CO, HC, and NO_*x*_ will be explored.

#### 4.2.1. CO Emission

**Figure 6:**
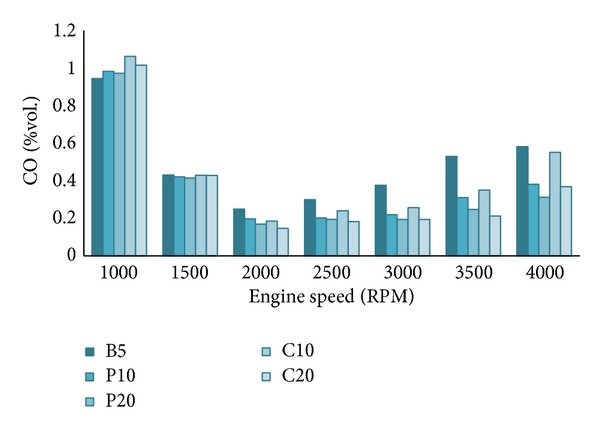
Carbon monoxide (CO) emission at full load.

CO emission is decreased with the increase of biodiesel content in the blends especially at higher engine speeds. From Figure 6, CO emission of any 20% blend is lower than that of the 10% blend. The oxygen rich biodiesels result in more complete combustion of the blends which helps to convert CO into CO_2_ [3, 32]. At higher engine speeds, the in-cylinder combustion temperature is high which results in more complete combustion. So, the trend is more dominant at higher engine speeds. At lower engine speeds, the higher kinematic viscosity of biodiesel predominates the combustion process which results in comparatively higher CO emission [30]. C20 blend showed lowest CO emission among the all tested blends. It can be attributed to the higher oxygen content of coconut biodiesel compared to others [18].

#### 4.2.2. HC Emission

**Figure 7:**
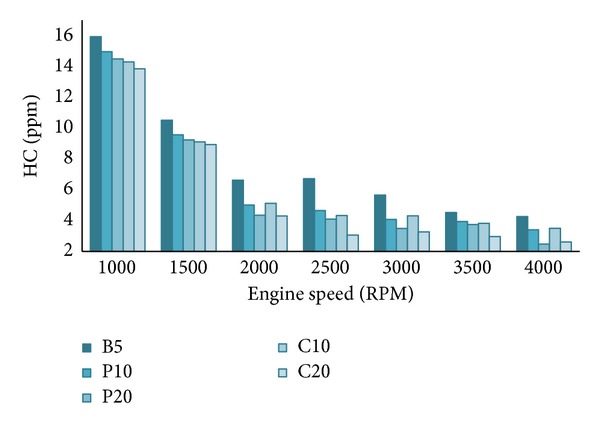
Hydrocarbon (HC) emission at 100% load.

Figure 7 shows the variations of HC emission for all the tested blends. The nature of HC emission is similar to CO emission. Here also the higher oxygen content of biodiesel is attributed for lower HC emission [4]. At lower engine speeds, the emission rate is high for all fuel blends but, at higher engine speeds, the trend is very much clear. According to Rahman et al. [4], higher engine speed ensures better mixing of fuel and air hence better combustion. And again, the 20% blends showed lower HC emission than the 10% blends and B5, throughout the whole speed range. Here again, the C20 blend showed lowest HC emission among the all tested blends.

#### 4.2.3. NO_*x*_ Emission

**Figure 8:**
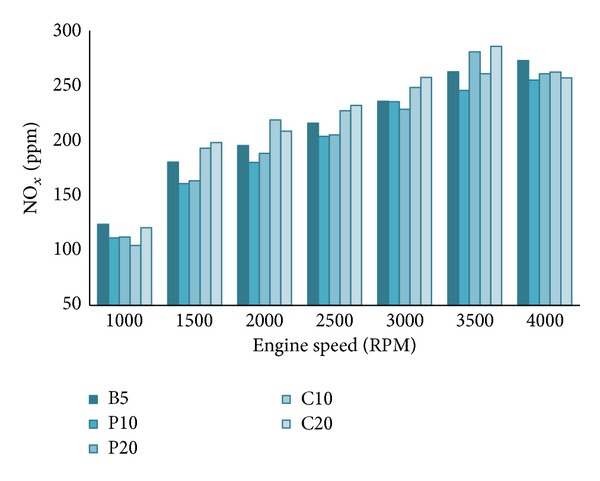
NO_*x*_ emission characteristics of all tested fuel blends at 100% load.

NO_*x*_ forms by the chain reactions involving O_2_ and N_2_ with the presence of sufficient temperature. So, the oxygen concentration and surrounding temperature are the key influence factors for NO_*x*_ emission. However, the engine load, speed, combustion chamber contents, homogeneity, and mixture density also have significant effects on NO_*x*_ emissions [33]. The general idea of NO_*x*_ emission is that it increases when the in-cylinder temperature is high. So, the high concentration of oxygen molecule in biodiesel promotes better combustion and hence higher NO_*x*_ emission. Again, the cetane number and viscosity also have influence on NO_*x*_ emission. Biodiesels exhibit higher cetane number and viscosity than the diesel fuel. Increment of biodiesel percentage in blends increases the cetane number and viscosity of the blends [3]. Higher cetane number leads to shorter ignition delay which allows the fuel-air mixture and the initial combustion products to have a longer residence time at elevated temperature and hence increases the thermal NO_*x*_ formation [4]. Kalam et al. [34] explained that the higher viscosity leads to a bigger droplet size and shorter ignition delay period which eventually increases NO_*x*_ emission. The combustion process improves when the speed increases due to more homogenous mixture of fuel and air. So, the NO_*x*_ emission increases with the increase of engine speed. From Figure 8, it can be said that NO_*x*_ emission increases with biodiesel blend ratio and engine speed. Palm blends (P10 and P20) have shown lower NO_*x*_ emission compared to other blends. It may happen due to the lower viscosity of palm blends compared to coconut blends. Most of the researchers reported similar trends of CO, HC, and NO_*x*_ emission in both single cylinder and multicylinder engine at similar engine conditions like ours.

### 4.3. Heat Loss Analysis

#### 4.3.1. Supplied Fuel Energy (*Q*_*s*_)

**Figure 9:**
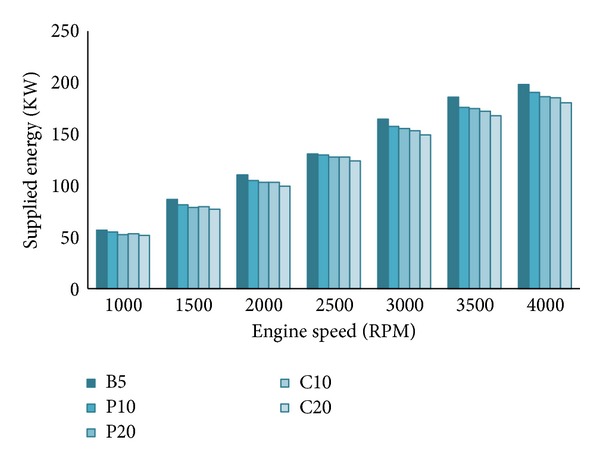
Supplied fuel energy variation.

Supplied fuel energy of all the tested blends are given in Figure 9. The supplied fuel energy (*Q*_*s*_) is the amount of fuel entering into the combustion chamber of an engine multiplied by its calorific value. Biodiesels have higher density and lower calorific value compared to fossil diesel [3]. The mass flow rate is higher for all blends, but the volume flow rate is the same since it is controlled by the fuel injector. Biodiesel increment lowers the supplied fuel energy of the blends as it lowers the calorific value [21]. The supplied fuel energy for B5 is the highest since its calorific value is the highest among the all tested blends. As the speed increases, more fuel is required to burn in the combustion chamber which means supplied fuel energy increases with increasing speed.

#### 4.3.2. Water Heat Loss (*Q*_*w*_) and Lubricating Oil Heat Loss (*Q*_oil_)

**Figure 10:**
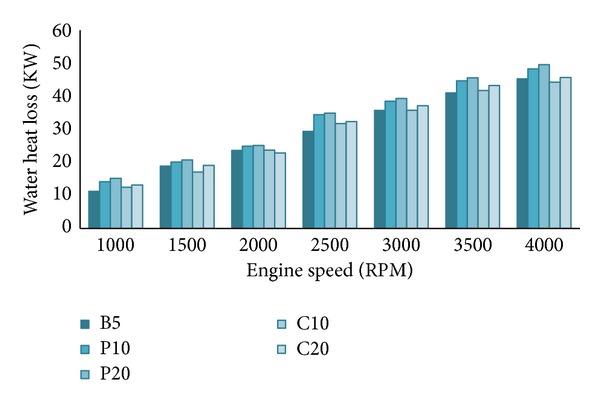
Water heat loss (*Q*_*w*_) variation at 100% load.

**Figure 11:**
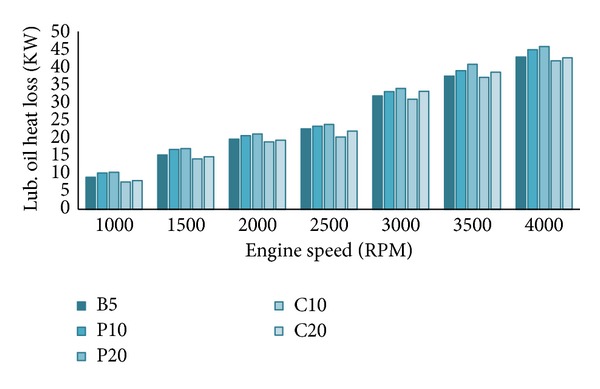
Lubricating oil heat loss (*Q*_oil_) for all tested blends at full load.

From Figures 10 and 11, both the water heat loss (*Q*_*w*_) and the lubricating oil heat loss (*Q*_oil_) are higher when biodiesel percentage increased in the blends at all RPMs. As explained earlier, biodiesel promotes better fuel combustion; hence the in-cylinder combustion temperature, pressure, and heat release rate are higher when the engine is running on biodiesels. So, the water heat loss and the lubricating oil heat loss increase about 1–3% and 1-2%, respectively, for 5% biodiesel increment in the blends. This will be clearer in the energy balance section. This trend of heat loss is supported by other researcher's results [22, 35]. We already know that with the increase of engine speed both the supplied fuel energy and the brake power increase. Similarly, all the heat losses also increase with engine speed, but the increment is not the same for all heat losses. Both heat losses are slightly higher for palm blends compared to other blends. It indicates that palm blends have higher combustion efficiency hence higher brake power compared to other blends which we have seen in Figures 3 and 5. Despite the higher heat losses, the palm blends (P10 and P20) showed higher brake power because this extraheat losses will be recovered by lower exhaust heat loss and unaccounted heat loss which will be discussed in the next section. Some researchers [2, 21, 33] have suggested providing ceramic insulation around the cylinder walls and head to reduce this two heat losses which eventually will increase the exhaust heat loss.

#### 4.3.3. Exhaust Heat Loss (*Q*_exh_)

**Figure 12:**
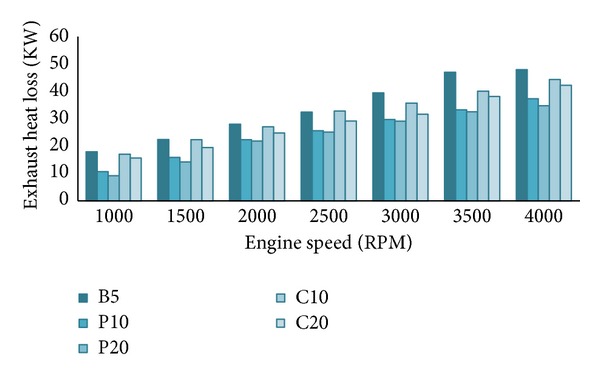
Exhaust heat loss (*Q*_exh_) at full load.

**Figure 13:**
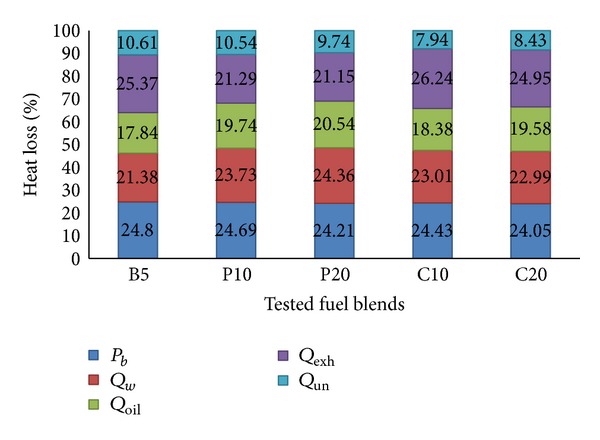
Engine energy balance at 2000 RPM and 100% load.

**Figure 14:**
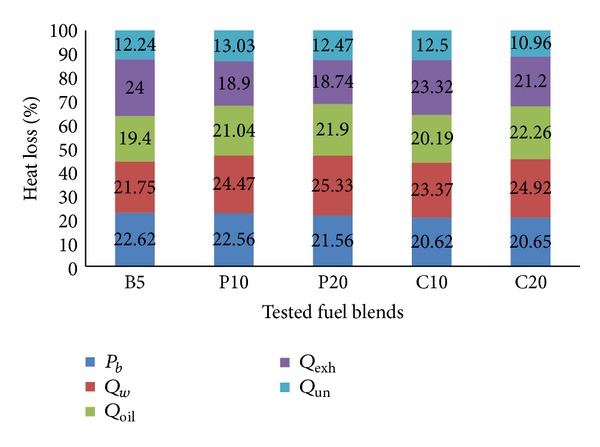
Engine energy balance at 3000 RPM and 100% load.

From Figure 12, B5 exhibits highest exhaust heat loss (*Q*_exh_) compared to other blends. It is already seen in the emission section that the palm blends give lower emissions compared to others and here they are showing lower exhaust heat loss. From the figure, it is also clear that biodiesel increment in the blends lowers exhaust heat loss. This trend of exhaust heat loss can also be explained from the exhaust gas temperature characteristics. Lower exhaust gas temperature signifies lower exhaust heat loss. We observed decreasing exhaust gas temperature trend with the increase of biodiesel percentage in our experiment. Similar trend of exhaust gas temperature was reported by the other researchers too [4, 27, 30]. The exhaust heat loss reduces by 0–2% when the biodiesel percentage increases in the blends from 10%. This will be more clear from Figures 13 and 14.

Finally, the unaccounted heat loss (*Q*_un_) is computed by subtracting the summation of all heat losses from the supplied fuel energy. It covers mostly the convection and radiation heat losses from the cylinder walls and also other unknown heat losses from the engine [2]. The trend of this heat loss is not definite. Similar results for unaccounted heat loss were presented by [24, 36].

#### 4.3.4. Energy Balance

The energy balance has been shown for all tested fuel blends in Figures 13 and 14 at 2000 and 3000 RPM, respectively. The percentage of heat losses have been calculated by dividing the individual heat losses with supplied fuel energy. Each column displays the distribution of the percentages of supplied fuel energy as brake power and heat losses for a specific fuel blend. This approach will provide a clear picture of the heat losses from the engine. For example, when the engine is operated with 10% palm (P10) biodiesel at 2000 RPM, 24.69% of the supplied fuel energy is converted to useful work, 23.73% is lost with the cooling water, 19.74% is lost through the lubricating oil, 21.29% is lost with the exhaust gas, and the remaining 10.54% is unaccounted heat loss. Now, if we compare the results with P20 at 2000 RPM, the brake power is decreased by 0.5%, the cooling water heat loss is increased by 0.63%, the lubricating oil heat loss is increased by 0.8% while the exhaust heat loss is decreased by 0.15%, and the unaccounted heat loss is decreased by 0.8%. If we compare P10 at 2000 RPM with P10 at 3000 RPM, we will see that the cooling water and lubricating oil heat losses are increased while the exhaust heat loss is decreased as explained before. We can also see that palm blends have shown lower exhaust heat loss and hence higher brake power compared to coconut blends, at any RPM. It has been possible due to the much lower exhaust loss of palm blends despite the higher cooling water and lubricating oil heat losses compared to coconut blends.

## 5. Conclusions 

The engine performance, emission, and heat loss analysis have been experimentally investigated using 10% and 20% blends of palm and coconut biodiesel in a multicylinder diesel engine. The theory of heat loss measurement, heat loss analysis, and a sample energy balance sheet showing the percentage of all heat losses have also been provided in this paper. However, based on our investigation, the following major conclusions have been drawn.

1. B5 showed highest brake power and lowest BSFC throughout the entire speed range compared to other blends. This is also reflected in the BTE curves. Palm blends, especially the P10 blend, showed very much similar performance. Coconut blends (C10 and C20) are the poorest among the all blends in terms of engine performance.
2. C20 blend showed lowest CO and HC emission whereas the palm blends (P10 and P20) showed almost similar result. In case of NO_*x*_ emission, the palm blends showed very good performance compared to other blends. With the increase of biodiesel percentage, NO_*x*_ emission increased for all tested fuel blends.
3. Both the water heat loss and lubrication oil heat loss increased with the increase of blend ratio, whilst the exhaust heat loss decreased. Here, the palm blends (P10 and P20) showed higher water and lubricating oil heat loss but, again, they showed lower exhaust heat loss which recovered their higher losses and eventually showed good performance compared to coconut blends.

At the end, we can say, if we go beyond B5, the brake power slightly falls but the CO and HC emissions improve a lot though the NO_*x*_ emission increases. Among the all tested blends, palm blends are more promising than others. If the higher water and lubricating oil heat losses of palm blends can be reduced by applying thermal barrier coatings (TBC) or ceramic coatings around the cylinder walls and head, then it may be possible to add a few KWs with the engine brake power.

## Figures and Tables

**Table 1:** Specification of the engine.

Parameter	Specification
Engine type	In-line four cylinder
Bore	91.1 mm
Stroke	95.0 mm
Displacement	2.5 L (2476 cc)
Compression ratio	21 : 1
Power	55 kW at 4200 rpm
Torque	142 Nm at 2500 rpm
Fuel injection system	Common rail mechanically controlled

**Table 2:** Measured fuel properties of all tested fuel blends.

Properties	B5	P10	P20	C10	C20	Testing equipment	Test method and specification limits
Density at 15°C (kg/m^3^)	834.6	838	849.5	839.4	849.1	SVM 3000 (Anton Paar, UK)	ASTM D4052
Calorific value (MJ/kg)	43.4	40.5	40.1	38.2	36.8	C2000 basic calorimeter (IKA, UK)	ASTM D240
Viscosity at 40°C (cSt)	3.809	4.031	4.183	4.570	4.70	SVM 3000 (Anton Paar, UK)	ASTM D445 (1.9–6.0)
Flash point (°C)	80.3	183.5	184	168.1	171.6	Pensky-Martens flash point-automatic NPM 440 (Norma Lab, France)	ASTM D93 (>130°C)

**Table 3:** Exhaust gas analyser specification.

Gas analyser	Emissions	Methods	Maximum limit	Accuracy
BOSCH BEA-350	CO	Nondispersive infrared	10% vol.	±0.001% vol.
	CO_2_	Nondispersive infrared	18% vol.	±0.001% vol.
	HC	Flame ionization detector	9999 ppm	±1 ppm
	NO_*x*_	Electrochemical transmitter	5000 ppm	±1 ppm

## Nomenclature

*C*: Specific heat (KJ/Kg*·*K)
$\dot{m}$: Mass flow rate (Kg/s)
*N*: Engine speed (rev/s)
*P*: Power (KW)
*Q*: Heat (KJ)
*T*: Temperature (K).

### Subscripts

*a*: Air
*b*: Brake
cv: Control volume
exh: Exhaust
*f*: Fuel
*g*: Gas
*s*: Supplied
*w*: Water
un: Unaccounted.

### Abbreviations

BSFC: Brake specific fuel consumption (g/KW*·*h)
BTE: Brake thermal efficiency (%)
B5: 5% palm biodiesel + 95% diesel
C10: 10% coconut biodiesel + 90% diesel
C20: 20% coconut biodiesel + 80% diesel
LPG: Liquid petroleum gas
LHV: Lower heating value (KJ/Kg)
P10: 10% palm biodiesel + 90% Diesel
P20: 20% palm biodiesel + 80% Diesel
IC: Internal combustion
RPM: Revolution per minute.
